# Hard-Object Feeding in Sooty Mangabeys (*Cercocebus atys*) and Interpretation of Early Hominin Feeding Ecology

**DOI:** 10.1371/journal.pone.0023095

**Published:** 2011-08-26

**Authors:** David J. Daegling, W. Scott McGraw, Peter S. Ungar, James D. Pampush, Anna E. Vick, E. Anderson Bitty

**Affiliations:** 1 Department of Anthropology, University of Florida, Gainesville, Florida, United States of America; 2 Department of Anthropology, The Ohio State University, Columbus, Ohio, United States of America; 3 Department of Anthropology, University of Arkansas, Fayetteville, Arkansas, United States of America; 4 Department of Social and Behavioral Sciences, Santa Fe College, Gainesville, Florida, United States of America; 5 Laboratoire de Zoologie, Université d'Abidjan-Cocody, Abidjan, Côte d'Ivoire; Illinois State University, United States of America

## Abstract

Morphology of the dentofacial complex of early hominins has figured prominently in the inference of their dietary adaptations. Recent theoretical analysis of craniofacial morphology of *Australopithecus africanus* proposes that skull form in this taxon represents adaptation to feeding on large, hard objects. A modern analog for this specific dietary specialization is provided by the West African sooty mangabey, *Cercocebus atys*. This species habitually feeds on the large, exceptionally hard nuts of *Sacoglottis gabonensis*, stereotypically crushing the seed casings using their premolars and molars. This type of behavior has been inferred for *A. africanus* based on mathematical stress analysis and aspects of dental wear and morphology. While postcanine megadontia, premolar enlargement and thick molar enamel characterize both *A. africanus* and *C. atys*, these features are not universally associated with durophagy among living anthropoids. Occlusal microwear analysis reveals complex microwear textures in *C. atys* unlike those observed in *A. africanus*, but more closely resembling textures observed in *Paranthropus robustus*. Since sooty mangabeys process hard objects in a manner similar to that proposed for *A. africanus*, yet do so without the craniofacial buttressing characteristic of this hominin, it follows that derived features of the australopith skull are sufficient but not necessary for the consumption of large, hard objects. The adaptive significance of australopith craniofacial morphology may instead be related to the toughness, rather than the hardness, of ingested foods.

## Introduction

The adaptive significance of australopith facial form is a critical inference for understanding the hominin radiation, and the role that hard-object feeding (durophagy) has played in early hominin evolution has been contemplated for decades [1]–[9]. Recently, a finite-element stress analysis of the skull of *Australopithecus africanus* has been interpreted as indicating that the critical resources in the diet of this early hominin were large (10–50 mm diameter), hard objects that were habitually processed using the premolars and subsequently masticated by the molars [10]. While concerns over the application of the mathematical model have been reviewed elsewhere [11], this interpretation was accompanied by inferences of the functional consequences of premolar enlargement and facial morphology. Specifically, premolar enlargement in early hominins has been held to indicate greater use of these teeth in biting or chewing [10], [12]–[13]. The emphasis on premolar biting was also hypothesized to require buttressing of the facial skeleton in *A. africanus*, with forward placement of the zygomatic root and presence of anterior pillars representing two important structural solutions. The attendant mechanical advantage that the forward placement of the zygomatic affords also limits gape. These observations led to the conclusion that hard objects ingested by *A. africanus* were too large to be crushed between opposing molars, and that the premolars were engaged for initial crushing, with the molars masticating the fragmented seeds. In this model of oral processing the premolars are recruited to shatter the hard outer coats of seeds, and the molars subsequently process the softer seeds and nuts within. This functional partitioning of premolars and molars has been invoked to explain the “absence of a strong hard-object microwear signal in the molars of many australopiths” [10, p 2127], presumably because the potential hard-object microwear signal is restricted to the premolars. Even so, a new model of microwear feature formation stipulates that hard objects processed orally need to be sufficiently small (<5 mm diameter) in order to leave an occlusal enamel microwear signature at all [14]. Consequently, under this hypothesized feeding strategy [10] and microwear formation model [14], dental microwear is expected to differ between premolars and molars owing to their different roles in feeding, and microwear complexity (e.g., heavy pitting) is expected to be reduced or absent on the premolars, since large objects are hypothesized to be incapable of producing high levels of microwear complexity, especially in worn teeth.

**Figure 1:**
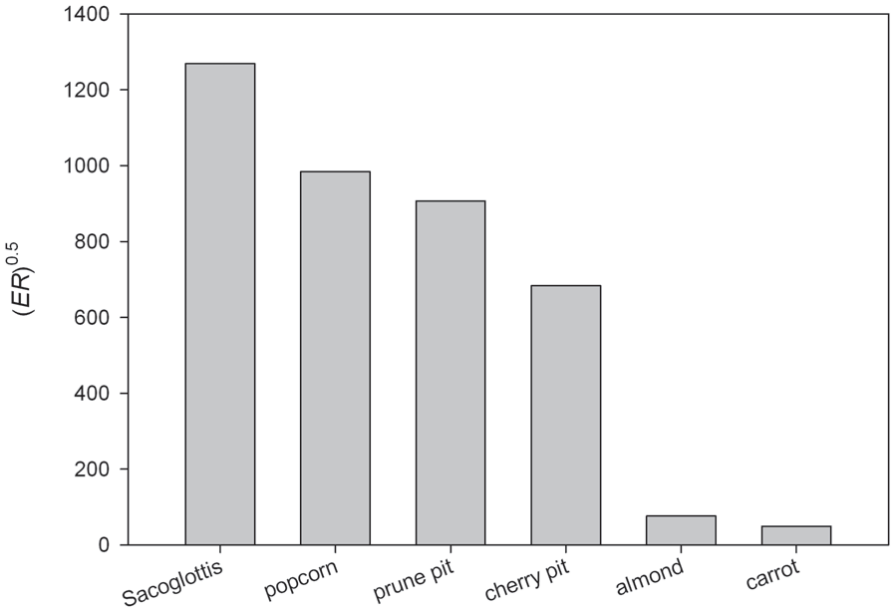
Fragmentation index of *Sacoglottis gabonensis* compared to familiar food materials. *E* is elastic modulus (MPa) and R is fracture toughness (Jm^−2^). High values indicate stress-limited foods, i.e., those expected to require high occlusal forces to induce structural failure. *Sacoglottis* is materially hard (high stiffness) but its “honeycombed” interior also renders it structurally tough. Comparative data are from ref 15.

Fortuitously, an extant cercopithecine monkey provides a suitable analog from which to test these predictions concerning the dietary adaptations of early hominins and augment our understanding of presumptively adaptive features related to durophagy in primates generally. The West African sooty mangabey, *Cercocebus atys*, is a terrestrial forager that habitually consumes the nuts of the fruit *Sacoglottis gabonensis* year-round, with seasonal fluctuations in which the nut comprises 25–80% of the monthly diet. The casing protecting the nut is highly stress-limited (fig. 1), as opposed to displacement-limited. Stress-limited foods fracture under high stress but low strain, whereas displacement-limited foods undergo relatively large deformations prior to crack formation [15]. Examples of stress-limited foods are items that are often described as “hard,” such as cherry pits and popcorn kernels, while displacement-limited foods, such as leaves, are described as “tough.”

**Figure 2:**
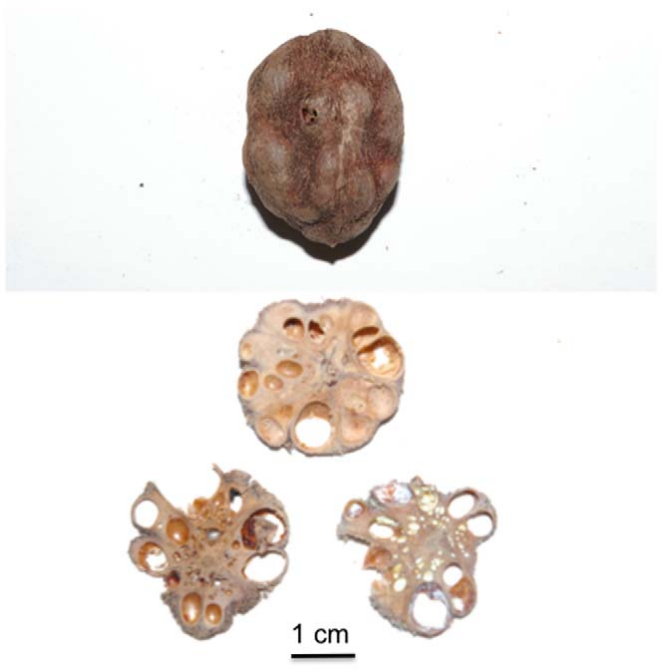
Seed casing of *Sacoglottis gabonensis* (top), and cross sections (bottom). Both the outer casing and the material comprising the inner compartments are hard, whereas the seeds found within the compartments (not pictured) are oily and relatively soft.

**Figure 3:**
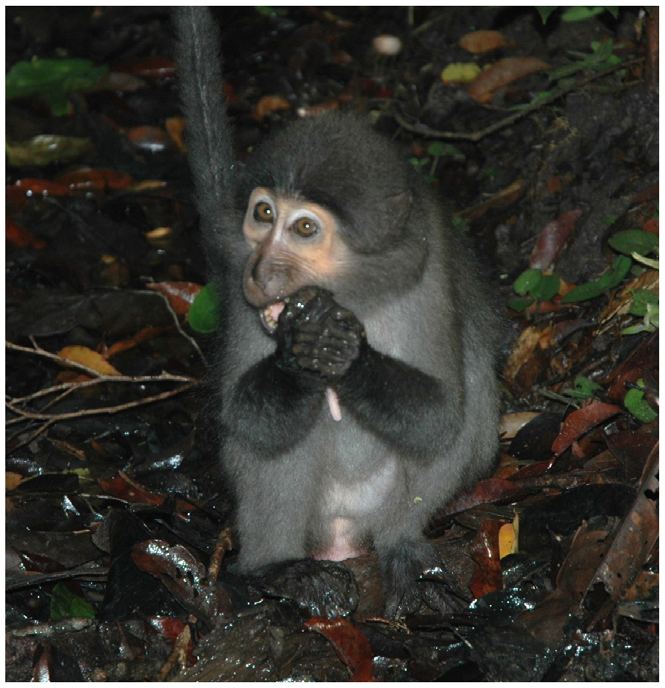
Sooty mangabey female processing *Sacoglottis gabonensis* in the Taï Forest, Côte d'Ivoire. Isometric biting with the postcanine teeth is an ingestive behavior associated almost exclusively (93.4% of all events) with *Sacoglottis*.

*Sacoglottis* provides a reasonable proxy for presumptive critical items of the *A. africanus* diet because in addition to being very hard ( = stress-limited), *Sacoglottis* casings are large (fig. 2), averaging 24 mm along their minor axis and 32 mm along their major axis (N = 9). Sooty mangabeys in the Taï Forest, Côte d'Ivoire process *Sacoglottis* in stereotypic fashion [16]: following manual harvesting from the leaf litter of the forest floor, the monkeys may scrape off any adherent material, and attempt to puncture the seed casing, all using the incisors. The casing is then placed behind the canines and one or more isometric bites are applied to shatter the object (fig. 3, Video S1). This is followed by expulsion of fragments and/or seeds from the oral cavity, a short bout of mastication, or placement in the cheek pouch for later processing.

We test predictions of large-object durophagy using *C. atys* as an analog for the inferred feeding behavior of *A. africanus* [10]. First, we examine whether enlargement of the premolars is consistently and exclusively associated with durophagy among anthropoid primates. Second, we argue that australopith facial morphology is sufficient but not necessary for large, hard-object feeding. Third, our examination of dental microwear tests predictions that microwear complexity is diminished with respect to feeding on large, hard objects. Collectively, these data challenge the hypothesis that facial morphology in *A. africanus* – and perhaps other, more derived australopiths – necessarily represents a specific adaptation to large-object durophagy.

## Results

### Premolar enlargement and durophagy

Enlargement of the second premolar (P4) relative to the molars distinguishes *Cercocebus* mangabeys and their sister taxon *Mandrillus* from other papionins [17], [18]. Comparative data [19] indicate that among living anthropoids, sooty mangabeys have large P4s relative to M1s (Tables 1, S1). Outside of the *Cercocebus- Mandrillus* group, the only taxa exceeding *Cercocebus atys* in the ratio of P_4_/M_1_ area are *Saguinus geoffroyi*, *Macaca nigra*, and *Pongo pygmaeus*. Of these taxa, only *Pongo pygmaeus* is a confirmed hard-object feeder, although they consume such stress-limited foods infrequently and are known to consume a variety of displacement-limited foods [20]–[21]. The high value for *Saguinus* is plausibly an effect of phyletic dwarfing [22]–[24], whereby it is a relatively diminutive molar – rather than an expanded premolar – that accounts for the extreme value. Dietary hardness is unknown in *Macaca nigra*, which in any case appears to feed infrequently on unripe fruit and seeds [25]. In these cases, and in comparison to other known durophagous taxa (*Cebus apella* [26], *Macaca nemestrina* [27], *Mandrillus sphinx* [28], *Cercocebus galeritus* [29] and *Cercocebus torquatus* [30]), the P_4_-M_1_ ratio in *Cercocebus atys* is not statistically significantly distinct. Its values are, however, significantly higher than *Lophocebus albigena*, another hard-object feeder [31]. While in general relatively large P_4_s are often found in hard-object feeders, not all durophagous primates have them, and there are taxa that have ratio values comparable to *Cercocebus atys* but do not engage in durophagy to any significant degree (e.g., *Hylobates lar*, *Gorilla gorilla*). Thus, premolar enlargement is associated with durophagy among living anthropoids, but not exclusively, and the relative degree of premolar expansion can vary greatly among hard-object feeders.

**Table 1:** Mean Ratio of P_4_/M_1_ area in *Cercocebus atys* and Higher Taxa.

Included Taxon	Ratio (P_4_/M_1_)
*Cercocebus atys*	0.8087
other *Cercocebus*	0.8449
subtribe Papionina	0.7775
tribe Papionini	0.7717
Cercopithecinae	0.7525
Cercopithecidae	0.7245
Catarrhini	0.7203
Anthropoidea	0.7240
Australopiths	0.7043

Data compiled from ref 19 except for *Cercocebus atys* which was derived from individuals collected under the Tai Monkey project (N = 8 females, 9 males); australopith data from refs 11,32. Other values represent the average of species means within higher taxa. Species ratio was calculated as the average of male and female means; these means were calculated as the mean P_4_ area/mean M_1_ area, with areas calculated as the product of mesiodistal and buccolingual dimensions.Mean ratio value for Anthropoidea includes australopith data. Mean values for individual taxa used in determination of included taxon values are provided in Table S1.

Enlarged, molarized premolars are frequently invoked as a functionally significant attribute of australopiths, particularly in *Paranthropus* [7], [10]–[11], [13], [32]. Relative to first molar size, however, australopith P4 size is unremarkable; in fact, the *Australopithecus* P_4_/M_1_ ratio is significantly less than that observed in *Cercocebus atys* (Tables 1, S1). In addition, the two *Australopithecus* species measured have P_4_s that are below what is expected (relative to M_1_ size) based on an anthropoid regression (i.e., their standardized residuals are negative, Table S1). This, of course, does not mean that early hominins did not possess large premolars; it merely underscores that the nature of premolar enlargement in modern primates (including hard-object feeders) is distinct from what is observed in australopiths. Premolar enlargement in early hominins was part of a general pattern of postcanine megadontia [9], [33]. That the enlarged P4s in *Cercocebus atys* are associated with hard food processing may not explain the molarization of premolars in australopiths, since in the latter case the concomitant expansion of the molars may signify adaptation to a different feeding strategy, including the processing of small, abrasive food items [5].

### Facial buttressing

As a papionin primate, *Cercocebus atys* possesses a craniofacial morphology more similar to *Macaca fascicularis* than *A. africanus*. *Macaca fascicularis* was contrasted to *A. africanus* in finite - element modeling to illustrate the difference in strain fields under premolar biting [10]. That the two models yield different strain patterns need not be questioned; however, our observations of feeding in the field empirically establish that a facial morphology not dissimilar to macaques is capable of withstanding the stresses routinely associated with large, hard-object feeding. We therefore suggest that from the standpoint of mechanical integrity, australopith facial morphology is sufficient, but not necessary, for the occasional or habitual processing of hard objects in the diet.

Sooty mangabeys lack both anteriorly-placed zygomatic roots and anterior pillars. In terms of mechanical advantage, *Cercocebus* mangabeys display a facial morphology that serves to maintain gape at the expense of maximizing bite force relative to sister taxa that consume hard objects [34]. As in the case of facial buttressing, bite force efficiency (the effective conversion of muscle force into bite force) is logically sufficient but not necessary for the habitual processing of large, hard objects.

Maximum bite force in *A. africanus* can be safely inferred, by virtue of body size differences alone, to have been much higher than in sooty mangabeys. Observation of mangabey feeding establishes their competence in habitual durophagy, and constitutes *prima facie* evidence that their dentition and jaws are competent for accommodating the attendant stress. Consequently, one need not suppose that maximization of bite force was a necessary adaptation among the australopiths for processing objects as stress-limited as *Sacoglottis*. *Cercocebus atys* manages to open these seed casings that under compressive stiffness testing yield at forces between 2000 and 3000 N. Since these figures exceed bite force capability in monkeys of this size [35], [36], it is clear that there are ingestive behavioral strategies that allow processing of these items in the absence of impossibly high bite forces. We suspect that during incision activities the sooty mangabeys are initiating cracks which, once subjected to isometric biting, grow quickly. This idea is supported by observations (N = 4,828) that sooty mangabeys discard 50.4% of *Sacoglottis* after initial incision; seed casings may be discarded because an effective broach was not achieved. It is also possible that at life stages when dental attrition has not progressed, the cusps of the postcanine teeth are used to produce point loads of the seed casing that can initiate cracks as well [37]. As attrition progresses, however, this strategy to initiate fracture becomes unavailable.

### Dental microwear complexity

**Figure 4:**
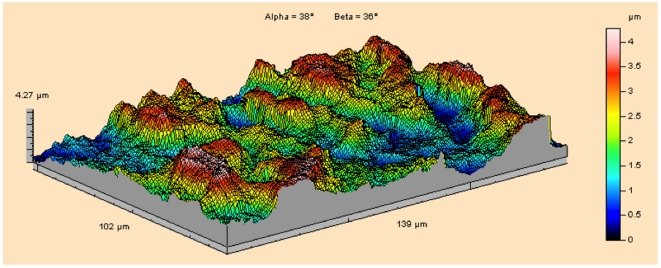
Enamel surface of the P^4^ of a *Cercocebus atys* specimen collected from Taï Forest, Côte d'Ivoire. The topography of wear resembles that of other primate hard-object feeders (e.g., some *Cebus apella* and *Lophocebus albigena*).

**Figure 5:**
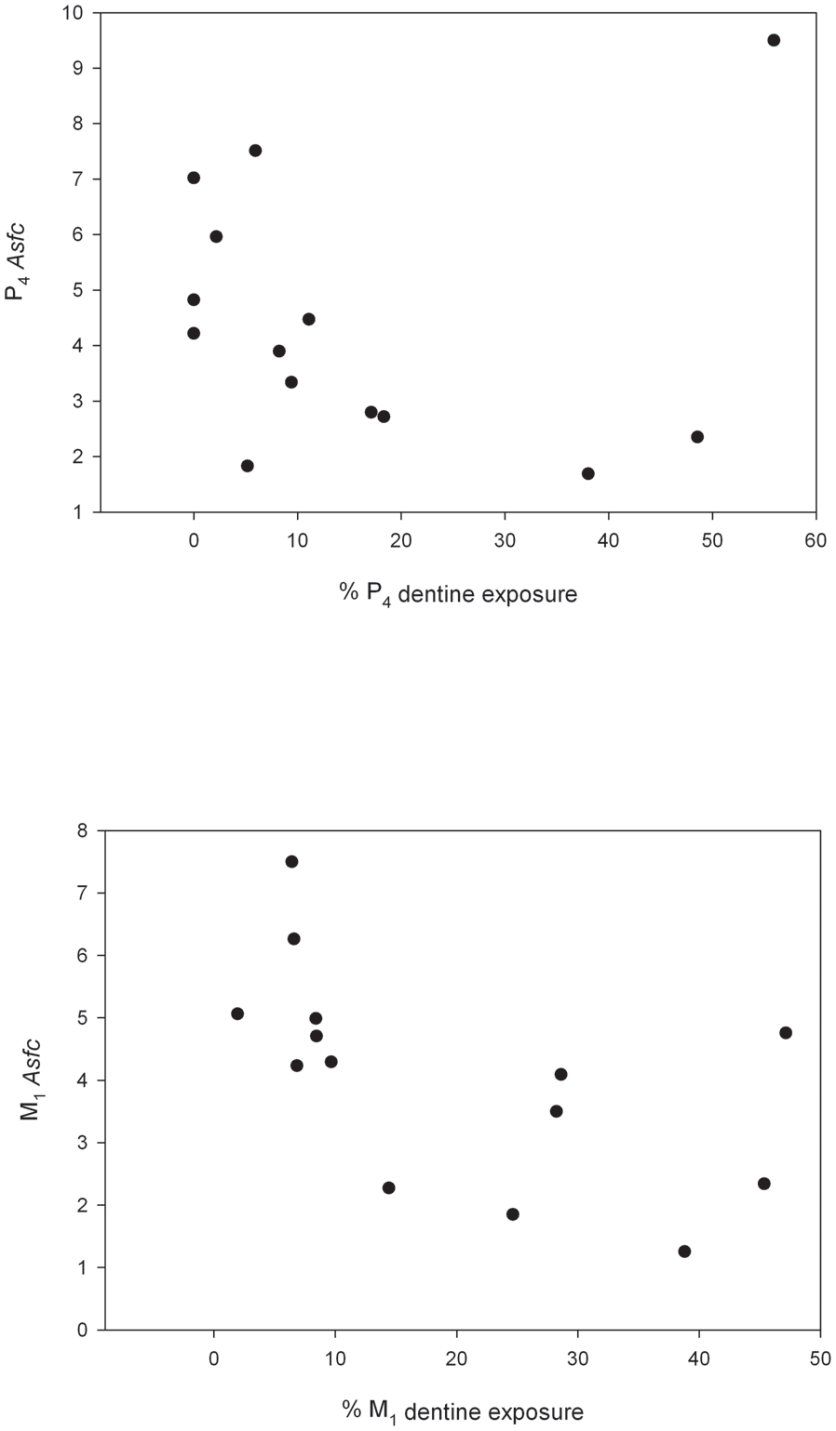
Relationship of microwear complexity to state of wear on P_4_ (*P* = 0.253 by Spearman's *ρ*) and M_1_ (*P* = 0.0081) in *Cercocebus atys*. Under an experimentwise *α* = 0.00625, none of the microwear texture indices covaries significantly with magnitude of attrition.

Our examination of P^4^ and M^1^ occlusal enamel indicates no significant difference between premolar and molar microwear fabrics (Table 2, Table S2), high microwear complexity on the premolars (fig. 4), and microwear texture that does not covary with attrition on either M^1^ or P^4^ (fig.5, Table S3). The *Cercocebus atys* microwear profile is highly complex in accord with those for other living hard-object feeders (Table 3). These results are potentially ascribed to the mechanical properties of the food objects themselves, the presence of grit adherent to the harvested nuts, or interaction between these factors. While grit may be expected to be adherent to the mesocarp of fruits harvested on the forest floor, the ingestive behavior of the Taï mangabeys suggests that this may not be the primary agent creating microwear. Sooty mangabeys at Taï routinely bite or scrape the seed casing using the incisors prior to placement of nuts on the postcanine tooth row: of the 5,029 examined bouts of feeding on *Sacoglottis*, only 185 (3.7%) involve premolar crushing in the absence of incisal action. Even if terrestrial foraging is plausibly implicated as a source of ingested grit, exogenous grit has been shown to exist high in the canopy [38], and many forest foods contain endogenous phytoliths likely capable of creating microwear. Hard-object feeders that are arboreal foragers (e.g., *Lophocebus albigena*) show complex microwear fabrics, and feature size and shape may be of value in distinguishing fabrics caused by abrasion with phytoliths versus exogenous grit [39].

**Table 2:** Dental Microwear Attributes of P^4^ and M^1^ in *Cercocebus atys*.

Variable	tooth	mean	st dev	skew	kurtosis	*P*
*Afsc*	P^4^	4.435	2.323	0.868	0.096	
	M^1^	4.076	1.726	0.135	−0.124	0.925
*Lsar*	P^4^	0.003	0.001	1.239	0.720	
	M^1^	0.004	0.001	0.576	−0.395	0.109
*Tfv*	P^4^	15873	1689.4	−0.105	2.541	
	M^1^	14834	2068.2	0.701	0.950	0.158
*Smc*	P^4^	27.267	53.139	2.865	9.084	
	M^1^	10.261	20.382	1.849	2.015	0.683

N = 14 for all samples. Two-tailed probabilities were evaluated via the Wilcoxon signed-ranks test. *Asfc* = area scale fractal complexity, describes “pittedness” of enamel surfaces; high values are observed in hard-object feeders among living primates.. *Lsar* = length scale anisotropy of relief, a measure of heterogeneity of the microwear fabric. *Tfv* = textural fill volume, a measure of the three-dimensional volume of smaller surface features. *Smc* = scale of maximal complexity, an indicator of feature size, with smaller features contributing to higher index values.

**Table 3:** Tests of Comparative Microwear Complexity (*Asfc*).

*Cercocebus atys* versus	difference	95% confidence interval	*P*
*Alouatta paliatta*	90.63	120.04–54.22	<0.001
*Cebus apella*	16.74	−18.06–51.54	0.844
*Lophocebus albigena*	41.16	7.58–74.74	0.005
*Pan troglodytes*	27.77	−4.84–60.38	0.162
*Pongo pygmaeus*	48.09	14.51–81.67	<0.001
*Trachypithecus cristata*	72.94	37.4–108.49	<0.001
*Gorilla gorilla beringei*	41.61	8.54–74.68	0.004
*Gorilla gorilla gorilla*	39.93	5.78–74.08	0.010

Probabilities based on Tukey's Honestly Significant Difference test for posthoc comparisons. ANOVA of *Afsc* among the nine taxa was significant at *P*<0.001. Difference values are based on rank-transformed data.

An assertion that microwear complexity differences among taxa are to some degree independent of food mechanical properties requires an assumption that among extant primates examined, hard-object feeders routinely encounter more grit on their ingested food than do those that do not eat hard foods. In any case, microwear likely reflects food fracture properties as such properties influence the approach of opposing occlusal surfaces to one another. Tougher, displacement-limited foods that are sheared involve more parallel approach, more striations and less complexity. Harder, stress-limited foods that are crushed and fail catastrophically involve more perpendicular approach, greater pitting and more complexity. Fracture characteristics of foods, consequently, condition the force vectors by which ingested particles interact with dental enamel and create wear. From this perspective, the microwear fabrics of durophagous primates are distinct from those ingesting more displacement-limited foods (e.g., leaves) because of distinctive dynamics of tooth-particle-tooth interactions during biting and mastication.

## Discussion

Functional inference of feeding adaptations in fossil hominins is theoretically straightforward but operationally challenging in terms of hypothesis testing. Neontological analogs for early hominin morphology and behavior have served to generate hypotheses of hominin paleoecology [1], [40]–[41] that could be subsequently tested by other, independent means [12], [42]–[44]. In addition, biomechanical analyses of the masticatory system have been undertaken to infer functional performance and feeding behavior [3], [8], [10], [45].

The logic of the use of modern analogs to draw paleontological inferences is transparent. Modern taxa whose morphological features converge on patterns observed in fossil taxa are assumed to engage in behaviors that, to some extent, characterized the extinct forms. In cases where the analog is distantly related to a fossil taxon, phylogenetic constraint must be assumed to be minimal if the validity of the analogy is to hold; moreover, any finding that the morphological complex in question is not associated with a particular function or behavior weakens the original analogy [46]; e.g., the hypothesis that thick molar enamel is an adaptation to durophagy is compromised by an observation of thin enamel in a hard-object feeder [47].

### The durophagous sooty mangabey as a living analog of early hominins

Accompanying the postcanine megadontia of early hominins is the presence of thick enamel on the molars [48]–[49]. These traits were also present in certain Miocene apes [9], and thick enamel in these forms has been postulated – by analogy to living primates – to be specifically linked to durophagy involving nut-cracking behavior [50]. Sooty mangabeys possess thick enamel on the premolars and molars that are used to crack *Sacoglottis* seed casings (Table 4). Among living primates, relative enamel thickness is greater in hard-object feeders [50]–[52], although thick molar enamel is not prerequisite to durophagy [47].

**Table 4:** Postcanine Enamel Thickness.

				Average Thickness	Relative Thickness 1	Relative Thickness 2
Source	Species	Tooth	N	Mean (sd)	Mean (sd)	Mean (sd)
This study	*Cercocebus atys*	M_3_	4	0.879 (0.112)	24.969 (1.977)	19.902 (3.030)
This study	*Cercocebus atys*	M_2_	3	0.826 (0.049)	24.552 (1.325)	19.664 (2.416)
This study	*Cercocebus atys*	P_4_	2	0.723 (0.077)	24.921 (1.642)	19.130 (1.628)
Ref 47	*Aotus trivirgatus*	M_1_	1	0.208	20.023	12.054
Ref 47	*Cacajao calvus*	M_2_	1	0.279	17.963	9.843
Ref 47	*Callicebus moloch*	M_2_	1	0.259	20.134	12.315
Ref 47	*Chiropotes satanas*	M_2_	2	0.223 (0.004)	18.559 (0.786)	10.353 (0.784)
Ref 47	*Pithecia monachus*	M_1_	1	0.276	19.779	11.81
Ref 47	*Cebus apella*	M_1_	2	0.500 (0.001)	24.909 (0.262)	18.845 (0.668)
Ref 51	*Cebus capucinus*	M_1_	3	0.75 (0.074)	22.537	15.13 (1.587)
Ref 51	*Lophocebus albigena*	M_1_	2	1.17 (0.013)	23.544	16.85 (0.530)
Ref 51	*Cercocebus torquatus*	M_1_	3	1.24 (0.118)	19.855	12.89 (1.659)
Ref 51	*Cebus apella*	M_1_	1	0.990	26.83	21.36
Ref 52	*Cebus apella*	M_2_	1	0.486	25.309	19.211
Ref 52	*Alouatta villosa*	M_3_	1	0.378	17.479	9.746
Ref 52	*Callithrix jacchus*	M_2_	1	0.110	15.214	8.109
Ref 52	*Saimiri sciureus*	M_2_	1	0.159	16.846	9.659
Ref 52	*Cercopithecus mona*	M_2_	1	0.379	18.335	11.294
Ref 52	*Erythrocebus patas*	M_2_	2	0.479	19.358	12.298
Ref 52	*Macaca mulatta*	M_2_	3	0.546	19.995	13.149
Ref 52	*Macaca mulatta*	M_3_	1	0.461	21.237	13.995
Ref 52	*Macaca nemestrina*	M_2_	1	0.732	21.712	17.177
Ref 52	*Macaca nemestrina*	M_3_	1	0.649	18.87	12.193
Ref 52	*Papio cynocephalus*	M_2_	1	0.598	19.426	12.442
Ref 52	*Theropithecus gelada*	M_2_	3	1.107	20.535	14.67
Ref 52	*Theropithecus gelada*	M_3_	3	1.261	22.641	18.186
Ref 52	*Presbytis cristatus*	M_2_	1	0.428	18.487	11.443
Ref 52	*Gorilla gorilla*	M_2_	1	0.827	16.095	8.791
Ref 52	*Pongo pygmaeus*	M_3_	2	0.985	22.204	11.651
Ref 52	*Pan troglodytes*	M_2_	1	0.725	19.005	11.219
Ref 52	*Pan troglodytes*	M_3_	2	0.823	21.649	14.699
Ref 52	*Homo sapiens*	M_2_	9	1.236	25.659	20.756
Ref 52	*Homo sapiens*	M_3_	4	1.468	27.557	23.845

Units in mm. Means and where available standard deviations presented along with three measures:

Average Thickness = Area of enamel cap÷Length of enamel dentine junction.

Relative Thickness 1 = (√Area of enamel cap÷Length of enamel dentine junction)×100.

Relative Thickness 2 = (Average Thickness÷√Area of the dentine)×100.

**Figure 6:**
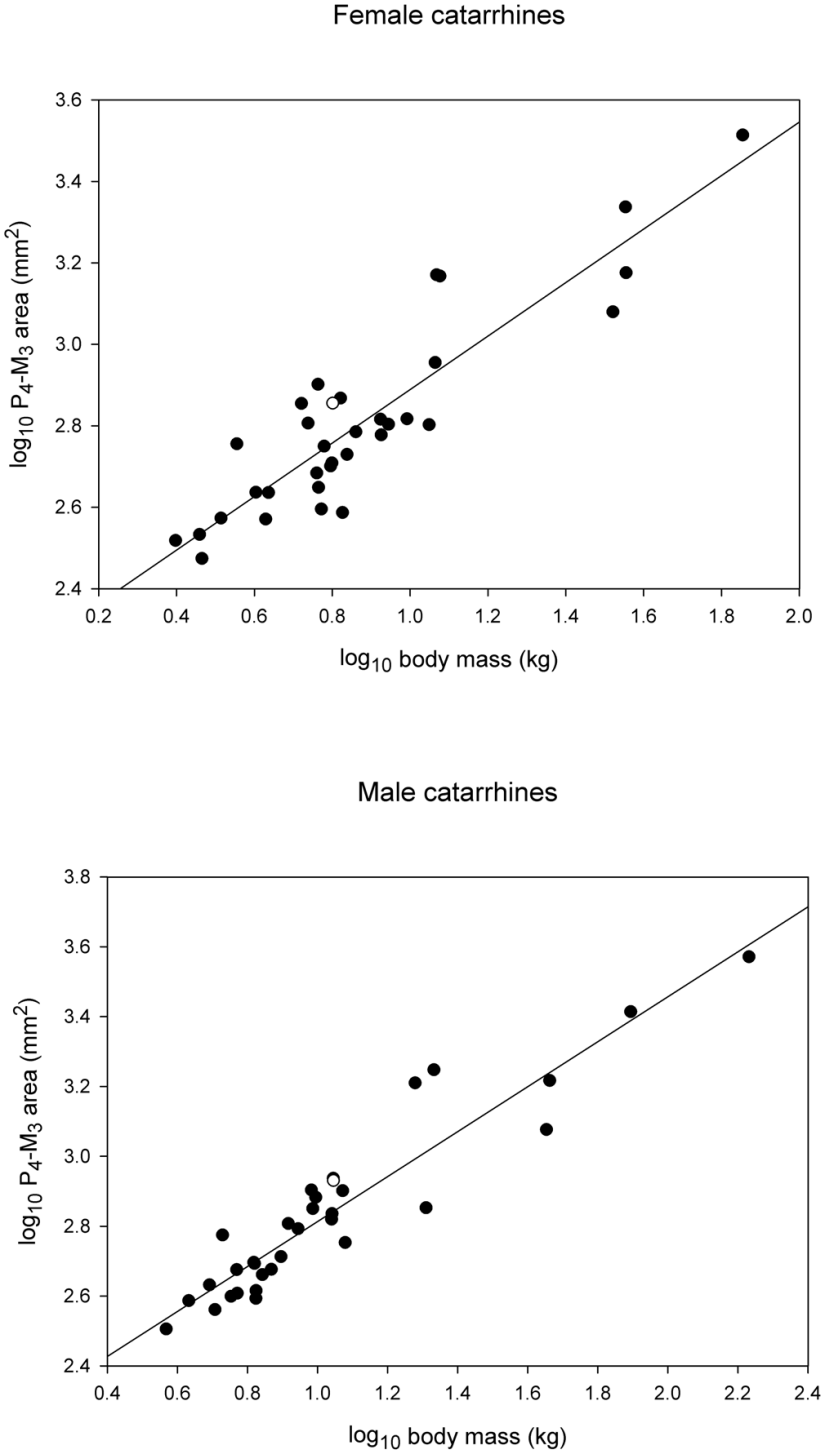
Relative tooth size in catarrhine primates (N = 33). *Cercocebus atys* is represented by open circles. Male and female sooty mangabeys have relatively but not exceptionally large postcanine teeth relative to body size. Tooth area data from ref 19; *C. atys* was measured from the Taï sample (N = 8 females and N = 9 males). Body weight data from refs 53,54.

There is no consensus on the significance of postcanine megadontia in early hominins with respect to durophagy. Megadontia is plausibly linked to diets that may have involved tough as well as hard foods [7], [45]. Comparative data [19], [53]–[54] suggest that sooty mangabeys display postcanine megadontia relative to body size (fig. 6), although they are not exceptional in this regard (Table S4). Taxa in which both males and females exceed *Cercocebus atys* in standardized residual values include only *Papio cynocephalus*, *Theropithecus gelada*, *Macaca fascicularis* and *Cerocebus galeritus*. Of these, *Cercocebus galeritus* is similar to *Cercocebus atys* in its durphagous habits [29], and though the diet of *Theropithecus* ostensibly involves hard objects [1], food toughness is perhaps as important a dietary challenge in this species [5]. Neither *Papio cynocephalus* nor *Macaca fascicularis* are known to be durophagous, yet both show greater expression of megadontia than *Cercocebus* mangabeys. The relationship of megadontia to diet is thus unclear and unpredictable.

Enlarged P4s, postcanine megadontia and thick molar enamel are plausibly linked to durophagy, although the processing of hard objects does not require this constellation of features. Collectively, these features can also be viewed as a means to limit attrition [55]–[56]. This explanation neither requires nor denies an important role for hard seeds and nuts as a source of attrition; ingested items across a spectrum of food types are known to produce wear [38].

### Mechanical trade-off of gape, effective bite force, and food size

On mechanical principles, primates are faced with the dilemma that gape can be maximized only at the expense of effective conversion of muscular force into bite force [57]. Gape is argued to be an integral constraint in the feeding behavior of *A. africanus*, limiting the initial fracture of hard objects to the premolars [10], because the benefit of enhancing adductor leverage is worth the cost of reducing gape. Gape is currently unknowable in fossil taxa, since the elasticity of the adductor mass – a function of pennation (the packing and orientation of muscle fibers) – is not recoverable. Gape at the premolars is estimated at 50 mm in *A. africanus* [10]. This figure places an upper limit on ingested food size, but also implies that means of extraoral food reduction were beyond the behavioral capacity of this species. Given living primate strategies for extraoral nut smashing [58]–[60], it is unreasonable to suppose that australopiths could not circumvent the problem of ingested food size. Both *Pan troglodytes* and *Cercocebus atys* endemic to Taï Forest, for example, occasionally process the hard nuts of *Coula edulis* in addition to those of *Sacoglottis*. The Taï chimpanzees, however, use hammerstones to process the nuts initially [58], while the mangabeys break the nuts entirely with their dentition. The chimpanzee strategy may be viewed as elective or obligate. It is unlikely that absolutely stronger chimpanzee jaws with their larger adductor mass could not process the nuts intraorally, even if chimpanzees have sensory mechanisms that discourages nut-cracking on thin-enamelled molars. The argument that *A. africanus* habitually and preferentially used their dentitions for initial reduction of large, hard objects [10] has important implications for their foraging behavior and behavioral ecology.

Even though hard-object feeding is associated with biomechanically robust mandibles [13], [20], [61], studies of adductor mechanical advantage do not invariably sort hard-object feeders from other species. Dietary toughness (emphasizing displacement-limited foods) is associated with improved mechanical advantage as much as is dietary hardness [26], [62]–[63]. Since improving mechanical advantage of the adductors will compromise gape, food size and geometry will covary with ingestive strategy independent of food mechanical properties to some degree [64]–[65]. *Cercocebus* species have apparently sacrificed bite force for gape; *Lophocebus* mangabeys, despite being described as “fallback” or seasonal hard-object feeders [31], are biomechanically better equipped to produce large occlusal forces in terms of craniofacial geometry [34].

Evolution of australopith facial form involves anterior migration of the zygomatic root, reduction in midfacial prognathism, and mediolateral expansion of the face, achieving extreme expression in *Paranthropus boisei* [3], [41]. One functional result of the first two features is greater mechanical efficiency of the jaw adductors; i.e., the more efficient conversion of muscular force into masticatory force. Among Cercopithecoidea, greater expression of these features is found among the Colobinae [13], [62], while *Cercocebus* mangabeys – being hard-object specialists – have craniofacial morphology that contrasts with the modern colobine condition. Since the colobine radiation is regarded as specializing on displacement-limited foods, the craniofacial skeleton of *Paranthropus* (and to a lesser extent, *Australopithecus*) is as plausible an adaptation to tough diets as to hard ones [7], [41], [45]. The strongest argument against this interpretation is the occlusal morphology and thick enamel that characterize australopiths as a group [5], [10].

### Microwear and dietary inference

The effective diameters of *Sacoglottis* seed casings are such that during postcanine crushing, initial structural fracture is likely to impact more than one isolated tooth in a given tooth row; this would also be true for *A. africanus* while feeding on objects as large as 50 mm across. The conjecture that premolar microwear would be distinct from that of the molars due to spatially limited food-tooth interactions makes intrinsic assumptions about the geometric regularity of ingested food that are unlikely to be met across all contexts. Analysis of premolar and molar microwear in *A. africanus* mirrors what is seen in *Cercocebus atys* in terms of overall similarity of microwear texture between teeth of the same individuals [11], with the difference that the higher complexity and reduced anisotropy in the microwear of *C. atys* is more clearly congruent with the microwear fabrics of other extant hard-object feeders.

The proximate causes of attrition and its microscopic detail are debated. Despite evidence that feeding on hard objects is associated with complex microwear fabrics [12], [42], [66], it is claimed that exogenous grit and only small, particularly hard food particles primarily account for microwear feature formation [10], [14]. That is, large objects are expected to be essentially invisible in terms of microwear. Interpretation of microwear in *A. africanus* and *Paranthropus robustus* as indicative of omnivory versus durophagy, respectively [2], [12], is thus challenged. Presumptive durophagy in *Paranthropus robustus* is reinterpreted as indicative of grit consumption analogous to terrestrially foraging baboons [67], while *Australopithecus africanus* is viewed as having the “expected” microwear signature of a seasonal hard-object feeder, albeit one that largely restricted its ingestion to large (>10 mm diameter) hard objects [10].

The anticipated finding of three microwear features has been offered as supporting a hypothesis of large, hard-object feeding in *A. africanus*: 1) different premolar and molar microwear patterns, owing to distinct use (initial fracture/crushing versus particle size reduction/fragmentation, respectively) during feeding, 2) absence of complexity features (i.e., “pits”) on premolar surfaces owing to their role in processing initially large nuts or seeds and 3) at late stages of attrition, diminished microwear complexity as the effects of large hard objects on occlusal enamel are reduced [10]. Using the sooty mangabey as a model for large, hard-object feeding, none of these features is observed in the context of large-object durophagy.

Interpretation of microwear fabrics is facilitated by recognizing that the amount of wear and the texture of the worn occlusal surface are not determined by identical agents. Abrasives are implicated in how much wear there is, but the texture attributes of the wear fabric depend on interactions of enamel occlusal surfaces and abrasives in or on food particles, on which local force vectors will have large effects. These vectors will vary according to the mode of fracture (i.e., the mechanical properties) of ingested foods. Thus, microwear complexity of sooty mangabeys is a function of the failure characteristics of *Sacoglottis*, and is not entirely dependent on the amount of ingested grit. The absence of similar complexity in *A. africanus* cheek teeth [11] is inconsistent with an interpretation of durophagy in this hominin taxon.

### Durophagy as a hominin adaptation

Our review of sooty mangabey craniofacial morphology in comparative context suggests that thick enamel is strongly associated with durophagy, while this feeding strategy is less clearly associated with megadontia and premolar expansion in living anthropoids. Moreover, the sooty mangabey presents a strong counterargument to the idea that durophagy is necessarily associated with comparatively efficient facial configurations for the production of bite force and stress mitigation. Furthermore, mangabey microwear suggests that durophagy was not the primary or fallback feeding adaptation in *A. africanus* In fact, microwear and stable isotope data in combination make a credible case for durophagy only in *Paranthropus robustus* [12], [44]; for East African australopiths, hard-object feeding does not appear to be the primary component of the diet [42], [68], [69]. Assuming dental hypertrophy is adaptive for resisting attrition – whatever the source – the derived mandibular and facial morphology is consistent with a loading environment that is high frequency, but not necessarily high magnitude [70]–[73]. In this view, the australopith skull may represent a primary adaptation for a low-quality diet requiring intensive and prolonged processing of fibrous, but not necessarily hard, foods. This interpretation is consistent with recent paleoecological scenarios which suggest that sedges and their underground storage organs were critical items in early hominin diets [44], [74], [75]. Tough foods present as significant mechanical challenges as hard foods [26], [76]. The objection to such an interpretation is that animals adapted to such diets display dental specializations (e.g., selenodont cheek teeth) that are completely unlike those of early hominins. Both material properties and geometry of foods are likely to be important determinants of dental form [64], [77], and a tough diet is not necessarily one composed of primarily two-dimensional foods (e.g., leaves) in which enamel blades and crests represent the optimal morphology for food breakdown. Nevertheless, the australopith dentition would appear to be suboptimal for processing a diet primarly composed of displacement-limited foods.

Under the assumption that hominoid precursors were bunodont and thick-enamelled [50], the most efficient occlusal solutions for a pronounced dietary shift may have been evolutionarily inaccessible to early hominins [56]. This invocation of phylogenetic constraint may be equivalently applied to *Cercocebus atys*: bilophodont molar crests might not represent the optimal occlusal morphology for nut-cracking, nor is this species' facial morphology optimal for producing the large forces required to do so. These observations underscore the inherent weakness of analogy for paleontological inference; that is, unidentified phylogenetic constraints in both the modern analog and the fossil form conspire to foil the comparison. The present exercise, however, is not entirely futile because the effects of large, hard-object feeding on the dentition have been examined, and the microwear complexity associated with this behavior is absent in the teeth of australopiths with the exception of *Paranthropus robustus*. With respect to *Australopithecus africanus* [10], the specific inference of durophagy is based, in part, on a theoretical analysis which revealed the early hominin cranium was “better designed to withstand premolar loads” than a cercopithecine model [10]. Yet since field observations establish that mangabeys are fully capable of sustained large-object durophagy, it becomes clear that the superior facial design for this activity in early hominins was not a requisite one.

On the other hand, facial features such as forward placement of the zygomatic root [13], orthognathic midface [62], large mandibular corpora [78] and wide faces and interorbital region [79] that describe modern colobines resemble what is observed in derived australopiths [3]. Whether these resemblances indicate functional convergence for dealing with displacement-limited foods is unknown, and in any case the same pitfalls of using extant analogies apply here as well. Fortunately, established methods that reflect actual ingestive events in the paleontological record – dental microwear and stable isotope analyses – provide glimpses into past behaviors unencumbered by the fog of evolutionary constraint. If we recognize the reality of such constraints, then findings of multiple and suboptimal morphological solutions to ecological problems may cease to surprise us.

## Materials and Methods

Diet and oral processing data on sooty mangabeys *Cercocebus atys* were collected from August 2008 to September 2009 in Taï forest, Côte d'Ivoire. The study group contains approximately 100 habituated individuals under continuous study since 1994. We used focal animal sampling to record all foods consumed by adults vs. non-adults of both sexes. For each food item consumed, we described the associated oral processing activities using four behavioral categories: 1) incising, 2) canine puncture, 3) post canine crushes (i.e., isometric biting), and 4) mastication cycles. We calculated the frequency that each oral processing activity occurred during a given focal period and the frequency with which each activity was associated with individual food species. Full description of sampling methods is provided elsewhere [16].

Mechanical testing of *Sacoglottis* was conducted on an MTS 858 system (Eden Prairie, MN). Elastic modulus was determined in compression on machined specimens (N = 5); toughness was determined from load-displacement curves from microtome guillotine application to prepared specimens (N = 8). All experiments were conducted under displacement control at a rate of 0.167 mm/s [15].

Attributes of dental microwear texture [43], [56], [80] were determined from *Cercocebus atys* specimens with sufficient enamel on both P^4^ and M^1^ “Phase II” facets to permit analysis. These teeth were collected opportunistically from naturally deceased individuals (N = 14) in Taï Forest, Côte d'Ivoire between 1994 and 2008. Original crowns were cleaned and molded with polyvinylsiloxane dental impression material, and casts were produced using a high resolution epoxy. Point clouds representing facet #9 surfaces were generated from replicas using a white-light scanning confocal profiler (Solarius Inc.) with a lateral sampling interval of 0.18 µm. Four adjacent fields of 138 µm×102 µm were sampled for a total area of 276 µm×204 µm.

Each point cloud was analyzed using ToothFrax and SFrax (Surfract Corp.) scale-sensitive fractal analysis software. Median values for each tooth of each specimen were computed for area-scale fractal complexity (*Asfc*), length-scale anisotropy of relief (*Lsar*), textural fill volume (*Tfv*), and scale of maximal complexity (*Smc*). These attributes are described in detail elsewhere [43]. Values for the premolars and molars of individuals were compared using Wilcoxon Signed-Ranks Tests [11], [56].

*Cercocebus atys* enamel thickness measures were obtained following existing procedures [47]–[48], [51]–[52]. Mandibular teeth (N = 2 P_4_, 3 M_2_, 4 M_3_) were cleaned and fixed using cyanoacrylate (to prevent chipping) and coronally sectioned through mesial and distal cusp pairs with a diamond-wafering blade on a Buehler-Isomet low-speed saw. Exposed sections were gently scoured with 0.5% phosphoric acid to enhance enamel-dentine boundaries. Digital photographs were processed in ImageJ [81] to obtain measures of tooth crown area, dentine area (DA), enamel cap area (EA), and enamel-dentine junction length (EDJ). Three values of enamel thickness were calculated [1]: average enamel thickness (EA/EDJ) [2], relative thickness 1 ([EA^0.5^/EDJ]×100) [3], relative thickness 2 ([average enamel thickness/DA^0.5^]×100).

Statistical evaluation of differences in P_4_/M_1_ area ratios utilized a bootstrap procedure. Dental metrics were resampled from the *Cercocebus atys* sample (with replacement) over 10,000 iterations to create a 95% confidence interval of the area ratio for comparison to other taxa, specific to the sample sizes used to calculate the area ratio for each species. In addition, we resampled the *Cercocebus atys* sample (with replacement) to create bootstrap means for comparison to mean values for other taxa. Bootstrap means were created using sample sizes specific to the taxon under comparison. Probability was determined as the number of bootstrap means in which the observed species mean value was matched or exceeded over 10,000 iterations. If, for example, the mean ratio for a taxon was less than that for the empirical mean for *Cercocebus atys*, probability was determined by counting the number of bootstrap iterations in which the resampled mean was as low or lower than that of the taxon under comparison.

## Supporting Information

Table S1 Data compiled from refs 18,19 (extant anthropoids) and refs 11,32 (australopiths), except for *Cercocebus atys* which was derived from individuals collected under the Tai Monkey project (N = 10 females, 8 males). We also collected data for the *Cercocebus chrysogaster* sample (N = 2 females, 7 males). Ratios were calculated as the average of male and female means; these means were calculated as the mean P_4_ area/mean M_1_ area, with areas calculated as the product of mesiodistal and buccolingual dimensions. Residuals calculated from regresson of log P_4_ area (Y) on log M_1_ area (X) from Model I (least squares) regression. The 95% confidence interval provided is based on a bootstrap estimate over 10,000 iterations in which the *Cercocebus atys* data are resampled (with replacement) at sample sizes reported for the taxon under comparison. Probabilities are calculated based on a bootstrap test for mean differences between *Cercocebus atys* and each taxon. This involves resampling the *Cercocebus atys* data over 10,000 iterations at sample sizes reported for the compared taxon. If the empirical mean for *Cercocebus atys* is greater than that of the compared taxon, what is tested is whether the resampled means are as small as or smaller than that of the compared taxon (and vice-versa if the sooty mangabey mean is less than that of the taxon being compared). Because there are 51 comparisons in all, we employ the Bonferroni correction to set α = 0.00098. A *P* value of zero indicates that in no case did the bootstrap means match or exceed the mean for the compared taxon.
(DOC)

Table S2 Asfc = area scale fractal complexity; Lsar = length scale anisotropy of relief; Tfv = textural fill volume; Smc = scale of maximal complexity.
(DOC)

Table S3 Under an experiment-wise significance threshold = 0.05/n comparisons, *α* = 0.00625. By this criterion, none of the microwear texture variables are correlated with attrition. Attrition was measured as the proportion of dentine exposed on occlusal surfaces relative to total crown area. Owing to distributional properties of microwear texture variables, Spearman's rank-order correlation was used for statistical evaluation.
(DOC)

Table S4 Tooth area data compiled from ref 19 except for *Cercocebus atys* which was derived from individuals collected under the Tai Monkey project (N = 8 females, 8 males). Body mass data from refs 53,54. Residuals calculated from regresson of log P_4_-M_3_ area (Y) on log body mass (X) from Model I (least squares) regression.
(DOC)

Video S1 An adult female sooty mangabey (*Cercocebus atys*) consuming a *Sacoglottis gabonensis* seed recovered from a swampy area in the Ivory Coast's Tai Forest. These seeds are the hardest items in the sooty mangabey diet and are also the most frequently consumed food item of all group members (with the exception of dependent young). Oral processing activities include incision and powerful, isometric bites both of which are readily apparent in this video.
(MPEG)
