# Supplementary material for: Hard-Object Feeding in Sooty Mangabeys (Cercocebus atys) and Interpretation of Early Hominin Feeding Ecology
Source: PLoS One. 2011 Aug 26;6(8):e23095. doi: 10.1371/journal.pone.0023095 (PMC3162570; doi:10.1371/journal.pone.0023095)
Supplement: Table S1 — Data compiled from refs 18,19 (extant anthropoids) and refs 11,32 (australopiths), except for Cercocebus atys which was derived from individuals collected under the Tai Monkey project (N = 10 females, 8 males). We also collected data for the Cercocebus chrysogaster sample (N = 2 females, 7 males). Ratios were calculated as the average of male and female means; these means were calculated as the mean P4 area/mean M1 area, with areas calculated as the product of mesiodistal and buccolingual dimensions. Residuals calculated from regresson of log P4 area (Y) on log M1 area (X) from Model I (least squares) regression. The 95% confidence interval provided is based on a bootstrap estimate over 10,000 iterations in which the Cercocebus atys data are resampled (with replacement) at sample sizes reported for the taxon under comparison. Probabilities are calculated based on a bootstrap test for mean differences between Cercocebus atys and each taxon. This involves resampling the Cercocebus atys data over 10,000 iterations at sample sizes reported for the compared taxon. If the empirical mean for Cercocebus atys is greater than that of the compared taxon, what is tested is whether the resampled means are as small as or smaller than that of the compared taxon (and vice-versa if the sooty mangabey mean is less than that of the taxon being compared). Because there are 51 comparisons in all, we employ the Bonferroni correction to set α = 0.00098. A P value of zero indicates that in no case did the bootstrap means match or exceed the mean for the compared taxon. (DOC) [file pone.0023095.s001.doc]

Table S1

Ratio of P_4_/M_1_ area in extant anthropoids

| Taxon | Ratio (P_4_/M_1_) | Standardized residual | Within 95% CI | Bootstrap *P* |
| --- | --- | --- | --- | --- |
| *Cercocebus atys* | 0.8087 | 1.113 | --- | --- |
| *Saguinus geoffroyi* | 0.8245 | 1.106 | * | 0.516 |
| *Aotus trivirgatus* | 0.6795 | - 0.714 |  | 0 |
| *Ateles geoffroyi* | 0.6395 | -1.123 |  | 0 |
| *Brachyteles arachnoides* | 0.6187 | -1.377 |  | 0 |
| *Cebus apella* | 0.8067 | 1.019 | * | 0.8238 |
| *Saimiri oerstedi* | 0.7887 | 0.639 | * | 0.1849 |
| *Saimiri sciureus* | 0.7509 | 0.219 |  | 0.0026 |
| *Alouatta seniculus* | 0.7257 | 0.126 |  | 0 |
| *Alouatta palliata* | 0.7504 | 0.405 |  | 0.0001 |
| *Cercopithecus cephus* | 0.7478 | 0.307 |  | 0.0083 |
| *Cercopithecus nictitans* | 0.7491 | 0.325 |  | 0.019 |
| *Cercopithecus mona* | 0.7352 | 0.141 |  | 0.0001 |
| *Cercopithecus mitis* | 0.7015 | -0.261 |  | 0 |
| *Cercopithecus neglectus* | 0.7350 | 0.171 |  | 0.0047 |
| *Cercopithecus ascanius* | 0.6936 | -0.421 |  | 0 |
| *Chlorocebus aethiops* | 0.6564 | -0.888 |  | 0 |
| *Lophocebus albigena* | 0.6768 | -0.566 |  | 0 |
| *Cercocebus torquatus* | 0.7838 | 0.825 | * | 0.1112 |
| *Cercocebus galeritus* | 0.7818 | 0.804 | * | 0.0979 |
| *Cercocebus agilis* | 0.8780 | 1.875 |  | 0.1117 |
| *Cercocebus chrysogaster* | 0.9360 | 2.445 |  | 0.0213 |
| *Macaca nemestrina* | 0.8058 | 1.089 | * | 0.7572 |
| *Macaca mulatta* | 0.6713 | -0.623 |  | 0 |
| *Macaca fascicularis* | 0.7245 | 0.059 |  | 0 |
| *Macaca nigra* | 0.8333 | 1.350 | * | 0.4208 |
| *Papio cynocephalus* | 0.6377 | -0.988 | * | 0 |
| *Theropithecus gelada* | 0.5737 | -2.125 |  | 0 |
| *Mandrillus leucophaeus* | 0.8063 | 1.155 | * | 0.4242 |
| *Mandrillus sphinx* | 0.9232 | 1.552 |  | 0.0097 |
| *Colobus polykomos* | 0.7409 | 0.289 |  | 0 |
| *Procolobus badius* | 0.7050 | -0.178 |  | 0 |
| *Nasalis larvatus* | 0.6541 | -0.886 |  | 0 |
| *Simias concolor* | 0.6770 | -0.579 |  | 0.0015 |
| *Pygathrix nemaeus* | 0.7037 | -0.190 |  | 0.0008 |
| *Rhinopithecus roxellana* | 0.5640 | -2.203 |  | 0 |
| *Trachypithecus phayrei* | 0.6700 | -0.671 |  | 0.0006 |
| *Kasi johnii* | 0.6193 | -1.422 |  | 0 |
| *Presbytis comata* | 0.7070 | -0.201 |  | 0 |
| *Trachypithecus cristata* | 0.6423 | -1.073 |  | 0 |
| *Hylobates klossi* | 0.6720 | -0.663 |  | 0.0002 |
| *Hylobates agilis* | 0.6695 | -0.683 |  | 0 |
| *Hylobates moloch* | 0.6710 | -0.686 |  | 0.0004 |
| *Hylobates lar* | 0.7850 | 0.769 | * | 0.3361 |
| *Pongo pygmaeus* | 0.8493 | 1.764 | * | 0.2683 |
| *Gorilla gorilla* | 0.7649 | 8.854 | * | 0.0759 |
| *Pan troglodytes* | 0.6301 | -1.092 |  | 0 |
| *Pan paniscus* | 0.6583 | -0.721 |  | 0 |
| *Australopithecus afarensis* | 0.6603 | -0.788 |  | 0 |
| *Australopithecus africanus* | 0.6770 | -0.388 |  | 0.0001 |
| *Paranthropus robustus* | 0.7395 | 0.506 |  | 0.0036 |
| *Paranthropus boisei* | 0.7545 | 0.746 | * | 0.0867 |

Data compiled from refs 18,19 (extant anthropoids) and refs 11,32 (australopiths), except for *Cercocebus atys* which was derived from individuals collected under the Tai Monkey project (N= 10 females, 8 males). We also collected data for the *Cercocebus chrysogaster* sample (N = 2 females, 7 males). Ratios were calculated as the average of male and female means; these means were calculated as the mean P_4_ area / mean M_1_ area, with areas calculated as the product of mesiodistal and buccolingual dimensions. Residuals calculated from regresson of log P_4_ area (Y) on log M_1_ area (X) from Model I (least squares) regression. The 95% confidence interval provided is based on a bootstrap estimate over 10,000 iterations in which the *Cercocebus atys* data are resampled (with replacement) at sample sizes reported for the taxon under comparison. Probabilities are calculated based on a bootstrap test for mean differences between *Cercocebus atys* and each taxon. This involves resampling the *Cercocebus atys* data over 10,000 iterations at sample sizes reported for the compared taxon. If the empirical mean for *Cercocebus atys* is greater than that of the compared taxon, what is tested is whether the resampled means are as small as or smaller than that of the compared taxon (and vice-versa if the sooty mangabey mean is less than that of the taxon being compared. Because there are 51 comparisons in all, we employ the Bonferroni correction to set α = 0.00098. A *P* value of zero indicates that in no case did the bootstrap means match the mean for the compared taxon.
