# Supplementary material for: Hard-Object Feeding in Sooty Mangabeys (Cercocebus atys) and Interpretation of Early Hominin Feeding Ecology
Source: PLoS One. 2011 Aug 26;6(8):e23095. doi: 10.1371/journal.pone.0023095 (PMC3162570; doi:10.1371/journal.pone.0023095)
Supplement: Table S2 — Asfc = area scale fractal complexity; Lsar = length scale anisotropy of relief; Tfv = textural fill volume; Smc = scale of maximal complexity. (DOC) [file pone.0023095.s002.doc]

Table S2. Dental Microwear Texture Analysis: *Cercocebus atys*

|  |  | *Asfc* | | *Lsar* | | *Tfv* | | *Smc* | |
| --- | --- | --- | --- | --- | --- | --- | --- | --- | --- |
| Specimen | Status | P4 | M1 | P4 | M1 | P4 | M1 | P4 | M1 |
| 2001 | subadult female | 2.715189 | 4.229522 | 0.005594 | 0.004812 | 16879.92 | 15802.44 | 43.73121 | 0.151936 |
| 2008 | adult female | 2.795621 | 4.087783 | 0.002292 | 0.001633 | 14559.41 | 12571.19 | 0.150668 | 0.208211 |
| 2010 | subadult male. | 9.499094 | 4.753032 | 0.00166 | 0.003275 | 16695.95 | 13975.57 | 0.15312 | 0.151773 |
| 2016 | subadult female | 3.893503 | 2.269858 | 0.002555 | 0.005688 | 15988.04 | 15955.95 | 27.86224 | 57.38742 |
| 2106 | adult male | 3.336482 | 4.291022 | 0.001469 | 0.004093 | 15580.19 | 14695.32 | 56.12747 | 29.07025 |
| 2108 | subadult male | 5.959175 | 6.259331 | 0.002161 | 0.002567 | 15515.39 | 14783.41 | 0.149933 | 0.150076 |
| 2138 | adult female | 7.508702 | 3.498321 | 0.001235 | 0.002566 | 16256.41 | 19639.51 | 0.151952 | 0.149992 |
| 2226 | subadult male | 4.468722 | 4.702636 | 0.001252 | 0.003279 | 15801.68 | 14518.19 | 7.588163 | 0.150966 |
| 2246 | subadult female | 4.819671 | 7.496143 | 0.00196 | 0.001498 | 16695.28 | 11671.03 | 47.70369 | 52.20024 |
| 243 | subadult female | 7.019302 | 5.059076 | 0.003274 | 0.002944 | 19637.13 | 13277.05 | 0.150265 | 0.150482 |
| 981 | adult female | 2.349804 | 2.339072 | 0.004965 | 0.003117 | 14219.81 | 16791.35 | 0.150074 | 0.150106 |
| 947 | subadult female | 4.216156 | 4.98493 | 0.002395 | 0.002662 | 16889.65 | 14663.15 | 197.3464 | 0.152115 |
| 949 | subadult male | 1.827727 | 1.845664 | 0.001661 | 0.006325 | 12064.28 | 12668.61 | 0.266792 | 0.267579 |
| 982 | adult female | 1.687556 | 1.253398 | 0.003772 | 0.00479 | 15439.93 | 16667.62 | 0.208756 | 3.313408 |

Asfc = area scale fractal complexity

Lsar = length scale anisotropy of relief

Tfv = textural fill volume

Smc = scale of maximal complexity
