# Supplementary material for: Hard-Object Feeding in Sooty Mangabeys (Cercocebus atys) and Interpretation of Early Hominin Feeding Ecology
Source: PLoS One. 2011 Aug 26;6(8):e23095. doi: 10.1371/journal.pone.0023095 (PMC3162570; doi:10.1371/journal.pone.0023095)
Supplement: Table S3 — Under an experiment-wise significance threshold = 0.05/n comparisons, α = 0.00625. By this criterion, none of the microwear texture variables are correlated with attrition. Attrition was measured as the proportion of dentine exposed on occlusal surfaces relative to total crown area. Owing to distributional properties of microwear texture variables, Spearman's rank-order correlation was used for statistical evaluation. (DOC) [file pone.0023095.s003.doc]

Table S3

Covariation of microwear texture and attrition in *Cercocebus atys*

| Tooth | Texture variable | Spearman’s *ρ* | *P* = |
| --- | --- | --- | --- |
| P^4^ | asfc | -0.328 | 0.253 |
| P^4^ | lsar | 0.199 | 0.494 |
| P^4^ | smc | -0.185 | 0.526 |
| P^4^ | tfv | -0.299 | 0.299 |
| M^1^ | asfc | -0.675 | 0.0081 |
| M^1^ | lsar | 0.204 | 0.483 |
| M^1^ | smc | -0.015 | 0.958 |
| M^1^ | tfv | 0.319 | 0.267 |

Under an experiment-wise significance threshold = 0.05/n comparisons, *α* = 0.00625. By this criterion, none of the microwear texture variables are correlated with attrition. Attrition was measured as the proportion of dentine exposed on occlusal surfaces relative to total crown area. Owing to distributional properties of microwear texture variables, Spearman’s rank-order correlation was used for statistical evaluation.
