# Supplementary material for: Hard-Object Feeding in Sooty Mangabeys (Cercocebus atys) and Interpretation of Early Hominin Feeding Ecology
Source: PLoS One. 2011 Aug 26;6(8):e23095. doi: 10.1371/journal.pone.0023095 (PMC3162570; doi:10.1371/journal.pone.0023095)
Supplement: Table S4 — Tooth area data compiled from ref 19 except for Cercocebus atys which was derived from individuals collected under the Tai Monkey project (N = 8 females, 8 males). Body mass data from refs 53,54. Residuals calculated from regresson of log P4-M3 area (Y) on log body mass (X) from Model I (least squares) regression. (DOC) [file pone.0023095.s004.doc]

Table S4

Relative Postcanine Megadontia in Living Catarrhines

Females

| Taxon | Ratio  (P_4_-M_3_ area/body mass^0.67^) | Standardized residual |
| --- | --- | --- |
| *Cercocebus atys* | 113.3 | 0.89724 |
| *Cercopithecus cephus* | 118.5 | -0.04502 |
| *Cercopithecus nictitans* | 87.4 | -0.75384 |
| *Cercopithecus mona* | 132.0 | 0.20931 |
| *Cercopithecus mitis* | 99.9 | -0.17079 |
| *Cercopithecus neglectus* | 107.8 | 0.03970 |
| *Cercopithecus ascanius* | 102.0 | -0.66222 |
| *Chlorocebus aethiops* | 114.5 | -0.00386 |
| *Lophocebus albigena* | 93.4 | 0.02428 |
| *Cercocebus torquatus* | 137.5 | 1.57981 |
| *Cercocebus galeritus* | 136.1 | 1.39954 |
| *Macaca nemestrina* | 111.3 | 0.89028 |
| *Macaca mulatta* | 72.3 | -0.49557 |
| *Macaca fascicularis* | 158.7 | 1.50706 |
| *Macaca nigra* | 117.0 | 0.82549 |
| *Papio cynocephalus* | 123.2 | 2.16687 |
| *Theropithecus gelada* | 126.6 | 2.24928 |
| *Colobus polykomos* | 78.0 | -0.24864 |
| *Procolobus badius* | 84.0 | -0.14826 |
| *Nasalis larvatus* | 66.8 | -0.66551 |
| *Simias concolor* | 77.9 | -0.53617 |
| *Pygathrix nemaeus* | 71.1 | -0.62531 |
| *Rhinopithecus roxellana* | 77.8 | 0.20519 |
| *Trachypithecus phayrei* | 81.2 | -0.49438 |
| *Kasi johnii* | 56.7 | -1.16486 |
| *Presbytis comata* | 57.6 | -1.82955 |
| *Trachypithecus cristata* | 83.9 | -0.48679 |
| *Hylobates klossi* | 66.6 | -1.40660 |
| *Hylobates agilis* | 76.5 | -0.85465 |
| *Hylobates moloch* | 80.4 | -0.54321 |
| *Pongo pygmaeus* | 60.8 | 0.85274 |
| *Gorilla gorilla* | 45.7 | 0.70183 |
| *Pan troglodytes* | 41.8 | -0.81484 |
| *Pan paniscus* | 36.2 | -1.56041 |

Males

| Taxon | Ratio  (P_4_-M_3_ area/body mass^0.67^) | Standardized residual |
| --- | --- | --- |
| *Cercocebus atys* | 76.8 | 0.95364 |
| *Cercopithecus cephus* | 90.0 | 0.08174 |
| *Cercopithecus nictitans* | 58.7 | -1.23626 |
| *Cercopithecus mona* | 71.3 | -0.75714 |
| *Cercopithecus mitis* | 65.8 | -0.60254 |
| *Cercopithecus neglectus* | 64.3 | -0.61652 |
| *Cercopithecus ascanius* | 86.5 | -0.37311 |
| *Chlorocebus aethiops* | 87.0 | 0.15888 |
| *Lophocebus albigena* | 77.8 | 0.50825 |
| *Cercocebus torquatus* | 67.5 | 0.43500 |
| *Cercocebus galeritus* | 83.3 | 1.10035 |
| *Macaca nemestrina* | 78.0 | 1.03060 |
| *Macaca mulatta* | 62.3 | -0.07735 |
| *Macaca fascicularis* | 111.0 | 1.50256 |
| *Macaca nigra* | 77.2 | 0.77961 |
| *Papio cynocephalus* | 82.4 | 2.46467 |
| *Theropithecus gelada* | 85.4 | 2.41754 |
| *Colobus polykomos* | 73.0 | 0.47825 |
| *Procolobus badius* | 70.5 | 0.13772 |
| *Nasalis larvatus* | 34.9 | -1.83975 |
| *Pygathrix nemaeus* | 60.2 | -0.24647 |
| *Trachypithecus phayrei* | 65.6 | -0.40823 |
| *Kasi johnii* | 47.2 | -1.27704 |
| *Presbytis comata* | 61.7 | -0.98937 |
| *Trachypithecus cristata* | 74.6 | -0.08124 |
| *Hylobates klossi* | 70.0 | -0.65981 |
| *Hylobates agilis* | 80.5 | 0.08814 |
| *Hylobates moloch* | 75.5 | -0.03140 |
| *Hylobates lar* | 68.7 | -0.68442 |
| *Pongo pygmaeus* | 33.2 | 0.27720 |
| *Gorilla gorilla* | 21.9 | -0.52297 |
| *Pan troglodytes* | 35.8 | -0.30398 |
| *Pan paniscus* | 26.5 | -1.87971 |

Tooth area data compiled from ref 1911,32 (australopiths)from refs ea includes lds)cope analysis of occlusal events. S Afr J Sci 77:203-230.xon.e consider ans exper except for *Cercocebus atys* which was derived from individuals collected under the Tai Monkey project (N= 8 females, 8 males). Body mass data from refs 53,54. Residuals calculated from regresson of log P_4_-M_3_ area (Y) on log body mass (X) from Model I (least squares) regression.
